# Removal of an Ovarian Tumor

**Published:** 1850-08

**Authors:** 


Dr. Van Buren, of New York, has lately removed successfully an ovarian tumor, of fibrous character, by the large abdominal section. The same surgeon has successfully performed amputation at the hip joint.

